# What is the impact of water sanitation and hygiene in healthcare facilities on care seeking behaviour and patient satisfaction? A systematic review of the evidence from low-income and middle-income countries

**DOI:** 10.1136/bmjgh-2017-000648

**Published:** 2018-05-09

**Authors:** Maha Bouzid, Oliver Cumming, Paul R Hunter

**Affiliations:** 1 Norwich School of Medicine, University of East Anglia, Norwich, UK; 2 Department of Disease Control, Faculty of Infectious and Tropical Diseases, London School of Hygiene and Tropical Medicine, London, UK

**Keywords:** systematic review, health systems, hygiene, maternal health

## Abstract

Patient satisfaction with healthcare has clear implications on service use and health outcomes. Barriers to care seeking are complex and multiple and delays in seeking care are associated with significant morbidity and mortality. We sought to assess the relationship between water, sanitation and hygiene (WASH) provision in healthcare facilities (HCF) and patient satisfaction/care seeking behaviour in low-income and middle-income countries. Pubmed and Medline Ovid were searched using a combination of search terms. 984 papers were retrieved and only 21 had a WASH component warranting inclusion. WASH was not identified as a driver of patient satisfaction but poor WASH provision was associated with significant patient dissatisfaction with infrastructure and quality of care. However, this dissatisfaction was not sufficient to stop patients from seeking care in these poorly served facilities. With specific regard to maternal health services, poor WASH provision was the reason for women choosing home delivery, although providers’ attitudes and interpersonal behaviours were the main drivers of patient dissatisfaction with maternal health services. Patient satisfaction was mainly assessed via questionnaires and studies reported a high risk of courtesy bias, potentially leading to an overestimation of patient satisfaction. Patient satisfaction was also found to be significantly affected by expectation, which was strongly influenced by patients’ socioeconomic status and education. This systematic review also highlighted a paucity of research to describe and evaluate interventions to improve WASH conditions in HCF in low-income setting with a high burden of healthcare-associated infections. Our review suggests that improving WASH conditions will decrease patience dissatisfaction, which may increase care seeking behaviour and improve health outcomes but that more rigorous research is needed.

**Key questions**

**What is already known?**

- A WHO/Unicef report (2015) highlighted the lack of adequate water, sanitation and hygiene (WASH) provision in many healthcare facilities (HCF) in low-income and middle-income countries (LMIC).
- Patient satisfaction and care seeking behaviour have been extensively used to monitor and improve the quality of care.
- The evidence on the contribution of poor WASH to patient dissatisfaction and care seeking behaviour is unclear.

**What are the new findings?**

- This systematic review sought to assess the relationship between WASH in HCF and patient satisfaction/care seeking behaviour in LMIC.
- Our findings showed that WASH status was not the main driver of patient satisfaction as other factors were more significant to users.
- Nevertheless, poor WASH provision was associated with significant patient dissatisfaction and stopped women from seeking care at maternity services.
- This is the first systematic review to be published on this topic.

**Key questions**

**What do the new findings imply?**

- Inadequate WASH provision in HCF in LMIC may increase the risk of healthcare-associated infections (HCAI).
- Beyond the HCAI burden, poor WASH provision may increase patient dissatisfaction and limit care seeking behaviour, leading to adverse health outcomes.
- Improving WASH provision in HCF should be prioritised as a means of addressing HCAI but also to address patient satisfaction and encourage timely care seeking.
- Global best practice guidelines combined with concerted action at the national policy level would support progress in ensuring adequate WASH provision in HCF in LMIC.

## Introduction

The water, sanitation and hygiene (WASH) attributable burden of disease is large and concentrated within low-income and middle-income countries (LMIC). A total of 842 000 diarrhoeal disease deaths (of which, 361 000 occurred in children under 5 years old) were attributed to inadequate WASH in 145 countries.^1^ Despite considerable progress in improving access to WASH services under the Millennium Development Goals (MDGs), a significant proportion of the world’s poor still lack access to safe WASH.^2^ However, reporting for the MDGs focused on WASH access in the community. By contrast, there has been little exploration of the impact of inadequate WASH provision in healthcare facilities (HCF) in LMIC. In 2015, WHO and Unicef assessed WASH status in 66 101 HCF in 54 LMIC.^3^ This assessment showed that 38% of facilities lacked access to water, 19% had no improved sanitation and 35% had no soap and water facilities. The issue of lack of WASH in HCF is of paramount importance because vulnerable populations are over-represented in these settings and the risk of infection and death is heightened. There is a growing awareness about this issue at a national and international level and an intergovernmental commitment to address this inequity. Indeed, progress on WASH provision in healthcare settings is currently being monitored as part of the Sustainable Development Goals (SDGs).^4–6^

Healthcare-associated infections (HCAI) are a major challenge in LMIC, where it has been estimated that the risk is 2–20 times higher than in developed countries.^7^ The highest rates of HCAI have been reported from the Eastern Mediterranean and South East Asia regions (11.8% and 10%, respectively) but this is an underestimation due to poor recording and lack of patient follow-up.^7^ As most HCAI are transmitted via the hands of healthcare workers through direct contact or environmental contamination, hand washing remains the single most important preventive strategy.^7 8^ The importance of WASH in healthcare settings was established long ago by the work of Alexander Gordon^9^ and Ignaz Semmelweis^10^ with regard to puerperal fever in the 18th and 19th centuries and more recently with regard to HCAI outbreaks where unsafe water or hygiene have been implicated.^11–14^ In contrast to high-income countries, there is relatively little evidence on the burden of HCAI in LMIC. A recent systematic review estimated that HCAI prevalence in LMIC was 15.5 per 100 patients, compared with 7.1 and 4.5 per 100 patients, in Europe and USA, respectively.^15^ It is plausible that much of this excess is due to inadequate WASH. However, the disease burden associated with inadequate WASH provision is likely greater than the HCAI burden alone. Indeed, inadequate WASH could have large impacts on health outcomes through its influence on patient satisfaction, care seeking behaviour and staff morale.

The barriers to care seeking are characterised using the three delays model developed by Thaddeus and Maine^16^ comprising: delays in deciding to seek care (primary delay), delays in reaching the health facility (secondary delay) and delays in receiving quality care once at the health facility (tertiary delay).^17^ Delays in receiving care have been estimated to be responsible for 30% newborn deaths in Uganda,^17^ 45% of child deaths from diarrhoea and acute respiratory infections in Mexico^18^ and an increased odds of intrauterine fetal death of 6.6 (95% CI 1.6 to 26.3) for over an hour delays among Women in Afghanistan.^19^

Care seeking barriers are multiple and include caretakers’ failure to identify early danger signs that should trigger appropriate care seeking behaviour, cost (especially for medication), distance to the facility, impediments related to weather or social unrest, lack of supervision for other children at home, lack of transport and, particularly relevant to this review, dissatisfaction with the quality of care.^20^ Afsana and colleagues^21^ consider that barriers to using hospital care are mainly related to care quality, especially for maternity services (often inadequate, unaffordable, insufficiently staffed and lacking medically trained professionals). Patient satisfaction is a commonly used indicator of healthcare quality and was shown to affect service use, clinical outcomes and patient retention.^22^ It is considered a reliable measure to understand patients’ needs and to make strategic decisions to improve care quality.^23^ However, no standardised system exists and a wide range of patient satisfaction indicators have been used as highlighted in a recent systematic review.^23^ The aim of this systematic review was to assess the impact of poor WASH provision in HCF in LMIC on two relevant indicators of healthcare quality: patient satisfaction and care seeking behaviour.

## Methods

The review methods are reported in accordance with the ‘Preferred Reporting Items for Systematic Reviews and Meta-Analyses’ (PRISMA)^24^ (checklist: online supplementary file 1).

10.1136/bmjgh-2017-000648.supp1 Supplementary file 1 sf1

### Search strategy and inclusion criteria

Pubmed and Medline Ovid were searched in March 2016 for articles published in English after the year 2000 using the search terms outlined in table 1. A combination of specific and broad search terms was used in order to retrieve all relevant papers. ‘Developing countries’ was included as a search term in two out of five searches so as not to exclude relevant studies. LMIC were classified based on income level as defined by the World Bank data. No restrictions on study design and duration were applied. Reference lists were manually scanned for additional relevant papers, which were included if eligible. Papers that had no WASH component were excluded.

**Table 1:** Combined search strategy and number of papers retrieved

Search strategy	Number of papers retrieved
(WASH OR Water OR Sanitation OR Hygiene) AND health care (MeSH: delivery of Health care) AND developing countries (Mesh) AND (satisfaction OR acceptance)	32
(water OR hygiene OR sanitation) AND care seeking AND developing countries	37
‘Patient Acceptance of Health Care’ AND (water OR sanitation OR hygiene)	461
Toilet AND (patient acceptance OR satisfaction)	87
Patient satisfaction AND developing countries	367
Total	984

### Data extraction and analysis

Relevant data were extracted from all included papers using a standardised form. These data were: geographic location, type of study, type of healthcare facility, intervention (if any) and main findings. All quantitative and qualitative findings were recorded. Data were summarised narratively and no meta-analysis was conducted because of the heterogeneity between studies and use of different indicators of patient satisfaction.

## Results

This systematic review assessed the effect of WASH in HCF on two quality of care outcomes: patient satisfaction and care seeking behaviour. Although WASH was rarely the primary focus of the included studies, all included some assessment of WASH conditions in HCF and their impact on patient satisfaction and/or care seeking behaviour.

**Figure 1:**
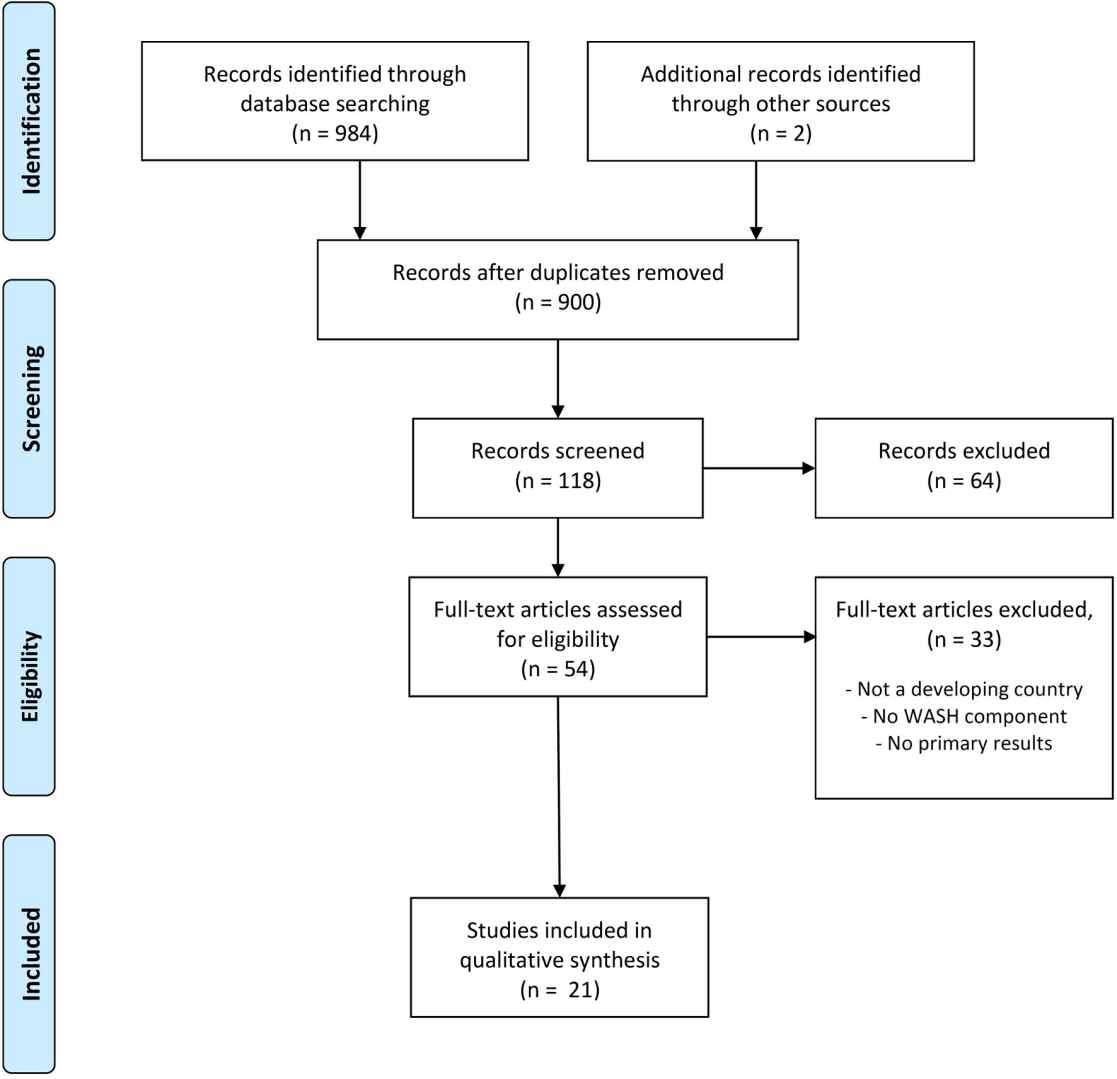
PRISMA flow diagram for peer-reviewed literature search and included studies. From Moher D, Liberati A, Tetzlaff J, *et al*. Preferred reporting items for systematic reviews and meta-analyses: the PRISMA statement. *PLoS Med* 2009;6:e1000097. For more information, visit www.prisma-statement.org.

The search strategy retrieved 984 articles (table 1). After removal of duplicates and screening of abstracts, 54 papers were considered eligible (figure 1). Following full text scanning, only 21 papers were found to have a WASH component and were therefore included. The details of the papers and extracted data are presented in table 2. Included papers covered various countries, settings and healthcare delivery systems. There were studies from India (n=4), Uganda (3), Ethiopia (2), Nigeria (2), Tanzania (2), Kenya (1), South Africa (1), Malawi (1), Burkina Faso (1), Madagascar (1) and Zambia (1). All but three studies were cross-sectional (18/21), with one case control study, two review studies and one systematic review.

**Table 2:** Impact of WASH in healthcare facilities on patient satisfaction and care seeking behaviour

Reference	Title	Location	Type of study	Context	Intervention	Findings	Further comments
**Water and sanitation**							
Woldeyohanes *et al*^25^	Perceived patient satisfaction with in-patient services at Jimma University Specialized Hospital, Southwest Ethiopia	Ethiopia	Cross-sectional	This study aims to measure and describe the level of patient satisfaction within in-patient healthcare services		**Toilet cleanliness: 18.5% (35/189) were satisfied while 81.5% (154/189) were dissatisfied.** 76.6% (145/189) were satisfied with cleanliness of the ward.	Research clearly identified a link between patient outcomes and patient satisfaction scores.
Tessema and Adnae^26^	Assessment of antiretroviral treatment (ART) care service provision in Tigray Region health centers, North Ethiopia	Ethiopia	Cross-sectional	Perceived levels of clients’ satisfaction with health services at ART clinic level in health centres		High scores of satisfaction were reported for courtesy and respect 95.80% (684/714) and privacy 93.28% (666/714). **Access and cleanliness to latrines were not always assessed. Toilet cleanliness was unsatisfactory for 35.32% (243/688).**	Adjusted OR for satisfaction was **2.22 (95% CI 1.62 to 6.32) for toilet cleanliness.** Measures such as increasing access to ART service, availing **clean toilet** and ART drugs may further increase client satisfaction. **Clean toilets are required especially for HIV/AIDS patients to prevent opportunistic and non-opportunistic coinfections.**
Tumlinson *et al*^33^	Quality of care and contraceptive use in urban Kenya	Kenya	Cross-sectional	The study hypothesis is that poor quality of family planning service provision is a barrier to contraceptive use		**78.5% of facilities (204/260) have running water.**	Facility infrastructure and most aspects of client satisfaction—including privacy issues, amount of information given, waiting time and overall satisfaction—were unrelated to contraceptive use.
Galukande *et al*^43^	Developing hospital accreditation standards in Uganda	Uganda	Cross-sectional	Accreditation is not well established in most developing countries for several reasons, including insufficient incentives, insufficient training and a shortage of human and material resources	Self-assessment hospital accreditation tool developed for a resource-limited context.	Among accreditation items (1) physical infrastructure and (2) infection control and waste management are relevant to WASH. 27.5% (11/40) hospitals were not tracking infection rates and 32.5% (13/40) had functional sterilisation equipment.	Good performance was measured in availability of equipment and **running water**, 24 hours staff calls systems, clinical guidelines and waste segregation. Poor performance was measured in care for the vulnerable, staff living quarters, physician performance reviews, patient satisfaction surveys and sterilising equipment.
Okwaro *et al*^44^	Challenging logics of complex intervention trials: community perspectives of a health care improvement intervention in rural Uganda	Uganda	Cross-sectional	Attract patients to health centres through improved services and attitudes of staff and better management of fevers	The intervention aims to enhance quality of care at public health centres and by extension improve malaria-related health indicators in community children	**Many health centres lacked running water and electricity.** Improvements in antimalarial drug availability were noted but community members were disappointed with the quality of care received. Patients continued to seek care at health centres they considered inadequate.	The intervention targeted malaria control to the exclusion of other diseases or **basic infrastructure such as in-patient facilities or clean water.** Requests by patients to increase the number of health workers, expand buildings and space within facilities, **provide clean water** and electricity, in-patient services, and **clean toilets** were reported.
Ezegwui *et al*^27^	Patients’ satisfaction with eye care services in a Nigerian teaching hospital	Nigeria	Cross–sectional	Evaluate patients’ satisfaction with the care received		**71.7% (220/307) were not satisfied with toilet facilities. There is only one toilet for patients and there is no running tap.**	The main areas of dissatisfaction were the cost of service **and toilet facilities.**
Khamis and Njau^30^	Patients’ level of satisfaction on quality of health care at Mwananyamala hospital in Dar es Salaam, Tanzania	Tanzania	Cross-sectional	Determine patients’ level of satisfaction on the quality of healthcare delivered at the out-patient department		422 patients were enrolled. Mean gap score was (−2.88±3.1) indicating overall dissatisfaction with the quality of care. Respondents were dissatisfied with general cleanliness (−0.50; p<0.001), and **sufficient chairs and toilets (−0.67; p<0.001).**	The questionnaire is divided into five dimensions (tangibles, reliability, responsiveness, assurance and empathy) to determine patients’ level of satisfaction. The mean gap score is calculated as the difference between mean perception score and mean expectation score.
Mohammed *et al*^31^	Assessing responsiveness of health care services within a health insurance scheme in Nigeria: users’ perspectives	Nigeria	Retrospective, cross-sectional survey	Insured users’ perspectives of their healthcare services’ responsiveness		42.8% (341/796) of users were satisfied with the quality of facilities. This included having enough space, seating places and fresh air in rooms and wards as well as a clean facility **and clean toilets in the hospital.** Low-income insured users reported better quality of facilities than high-income users (p<0.001).	Responsiveness is included in patient satisfaction and quality of care literature, and refers to the way individuals are treated and the environment in which they are treated. ‘Quality of basic facilities’ (**clean waiting rooms, toilet facilities,** examination rooms and surroundings) is important to patients in their experience of responsiveness.
Ray *et al*^28^	An assessment of rural health care delivery system in some areas of West Bengal-an overview	India	Cross-sectional observational study	Identify extent of utilisation of healthcare facilities and understand healthcare seeking behaviour in the community		**13.97% (63/451) were dissatisfied with care quality. 27% and 23% clients reported that toilets were ‘not at all usable’ and ‘dirty needing cleaning’,** respectively (n=174). **Safe drinking water was available in 55% of the facilities (n=18). Restrooms were either of poor quality or the clients did not use them, while they were not available in 3% of health facilities.**	Cleanliness of the premises, face-lift (of public health centres), **and clean toilet** with privacy and **availability of safe drinking water** could improve client satisfaction in rural healthcare delivery systems.
Sudhan *et al*^29^	Patient satisfaction regarding eye care services at tertiary hospital of central India	India	Descriptive study	To evaluate patients' satisfaction regarding eye care services		**The majority of respondents were highly satisfied with toilet 83.2% (133/160), water facilities 99.4% (159/160)** and cleanliness (159/160). **16.9% (27/160) did not answer the toilet question and one each for water** and cleanliness question.	
Westaway *et al*^32^	Interpersonal and organizational dimensions of patient satisfaction: the moderating effects of health status	South Africa	A cross-sectional analytical study design	To identify the underlying dimensions of patient satisfaction in diabetic clinic for black patients		263 patients were surveyed. The most important items for satisfaction were availability of a seat in the waiting area (0.73), **availability of a toilet in the waiting area (0.70)** and cleanliness (0.70).	Amenities and attributes of care were central to the organisational dimension of patient satisfaction. **Given lengthy waiting times in South Africa’s public health facilities, it is not surprising that the availability of a seat and toilet in the waiting area featured so prominently.** Cleanliness was also perceived as an important satisfaction area.
Glick^34^	How reliable are surveys of client satisfaction with healthcare services? Evidence from matched facility and household data in Madagascar	Madagascar	Cross-sectional	Investigation of the reliability of exit surveys by comparing patient satisfaction outcomes to population-based household surveys		An appearance index (mean of binary indicators for dirtiness, humidity damage, decay of walls, floors and ceilings, evidence of insects and **condition of toilet facilities (presence and cleanliness))** was calculated. The appearance index was 0.84 in household surveys and 0.91 in exit surveys for the same facilities. The number of respondents were 262 and 770, respectively.	The findings suggest that reported satisfaction in exit surveys is biased strongly upward for subjective questions regarding treatment by staff and consultation quality, but **not for relatively objective questions about facility condition** and supplies.
**Water, sanitation and hygiene in maternity services**							
Srivastava *et al*^35^	Determinants of women’s satisfaction with maternal health care: a review of literature from developing countries	Developing countries	Systematic review	Identify determinants of women’s satisfaction with maternity care in developing countries		Good physical environment was significant in women’s positive assessment of the health facility and maternal care services. In Bangladesh, mothers who rated the availability of services at the facility (a composite of waiting area, **drinking water, clean toilet** and waiting time) as ‘good’ were significantly more satisfied with care than those who rated the services as ‘poor’. Cleanliness, good housekeeping services and **maintenance of hygiene** were reported as determinants of satisfaction in Bangladesh, Gambia, Thailand, India and Iran.	Determinants of maternal satisfaction covered all three dimensions of care: structure, process and outcome. Structural elements included good physical environment, cleanliness, and availability of adequate human resources, medicines and supplies. Access, cost, socioeconomic status and reproductive history also influenced perceived maternal satisfaction. Process of care dominated the determinants of maternal satisfaction. Interpersonal behaviour was the most widely reported determinant, particularly around provider behaviour in terms of courtesy and non-abuse.
Steinmann *et al*^36^	Availability and satisfactoriness of latrines and hand washing stations in health facilities, and role in health seeking behavior of women: evidence from rural Pune district, India	India	Cross-sectional/questionnaire-based	Investigation of the WASH infrastructure in small health facilities and survey of expectations and satisfaction among women		**12 health facilities were assessed (6 private and 6 public). The mean number of latrines per healthcare facility was 2.4 (range 0–8), but was lower in public (mean 1.3; range 0–2) than in private facilities (mean 3.5; range 1–8). One facility had no latrine and one had an unimproved latrine.** **Generally, one hand washing station (tap) was available per latrine but two public facilities did not have any hand washing stations. The mean number of hand washing stations was 0.8 (range 0–2) in public facilities and 3.7 (range: 1–8) in private facilities.** **Soap was often missing from hand washing stations (6/12). Dedicated latrines for women were rare.**	**WASH installations in health facilities are generally acceptable in private facilitieswhile improvements are needed in some government facilities.** **Women expect WASH provision in health facilities, and view their quality in a broader framework of ‘cleanliness’, which they consider when choosing facilities.** **Key WASH features important to women are: number of latrines, their cleanliness and availability of water and accessories (such as dustbins).** Other factors, such as a good reputation, well-respected and competent doctors were considered more important than WASH status. **For ambulatory visits, including child birth, WASH status was seen as less critical than for prolonged hospitalisation.**
Philibert *et al*^42^	No effect of user fee exemption on perceived quality of delivery care in Burkina Faso: a case-control study	Burkina Faso	A quasi-experimental design with both intervention and control groups	Assessing whether women’s satisfaction with delivery care is influenced by a total fee exemption	In the intervention group, delivery care is free of charge at health centres	870 women were interviewed. 600 in intervention group and 270 in control group. 90% were satisfied with delivery care in both intervention and control groups. The poorest women were more highly satisfied with delivery environment than the wealthiest ones, especially concerning **hygiene** and comfort.	Quality of care was assessed using three components: care provider-patient interaction, nursing care and delivery environment. Patients are often inclined by courtesy to respond positively to questions on satisfaction with care quality. This level of courtesy is higher for interpersonal relationships between care providers and patients. Other biases: intimidation by male interviewer and non-sampling of remote households.
Mbwele *et al*^37^	Quality of neonatal healthcare in Kilimanjaro region, northeast Tanzania: learning from mothers’ experiences	Tanzania	Cross-sectional study using qualitative and quantitative approaches	Assess mothers’ experiences, perception and satisfaction with neonatal care in the hospitals		80 mothers were interviewed from 13 peripheral facilities and 32 from a referral hospital. **Only 2% discussed issues of hygiene**. One mother mentioned that the facility should **‘increase the level of hygiene’.** **The state of toilets at referral hospital were as expected for 59% respondents while, in peripheral hospitals 28% were as expected. Toilets were worse than expected for 7% and 26% in referral and peripheral hospitals, respectively.**	The most common reasons for primary delays: quality of treatment at the facility 55.1% (27/49) and cost of medical care 32.6% (16/49). Parameters for secondary delays were distance from home (11.1%) and combined distance and transport (7.4%).
Tetui *et al*^38^	Quality of Antenatal care services in eastern Uganda: implications for interventions	Uganda	Cross-sectional	Assessment of quality of ANC (Antenatal care) services in eastern Uganda with a goal of benchmarking		74.6% (217/291) respondents rated the ANC service as satisfactory. Infection control was available in 73.4% (11/15) facilities. Cleanliness was dissatisfactory for 4.1% (12/291), fairly satisfactory for 25.8% (75/291) and satisfactory in 70.1% (204/291).	Data collected to gauge infection control: **existence of piped running water, water buckets or basins, hand washing soap,** disposable hand drying towels, waste bins, sharps containers, disposable latex gloves and disinfection solution. The variables associated with high satisfaction were provider’s attitude (87.6%) and examination room privacy (83.5%). However, availability of medicines (32.3%) and waiting time (25.1%) had the highest dissatisfaction rates.
Gabrysch and Campbell^40^	Still too far to walk: literature review of the determinants of delivery service use	Low or middle income countries	Literature review (of review articles)	Identification of various factors related to delivery service use		Shortcomings in medical care are often coupled with shortcomings in hygiene. **Women criticise dirty toilet facilities, lack of water and aseptic practices** as well as lack of drugs or too early caesarean sections.	Perceived quality of care has an important influence on care seeking behaviour. Poor personal and medical quality of care, clash with culture and fear of procedures may decrease use.
Kongnyuy *et al*^45^	Criteria-based audit to improve women-friendly care in maternity units in Malawi	Malawi	Cross-sectional/interviews	To assess and improve women-friendly care in maternity units in Malawi	280 women were interviewed about care quality. The audit results were presented, and recommendations made. A re-audit (367 women) was conducted 3 months later and performance compared.	Significant improvement was recorded for cleanliness of maternity wards (89.6 vs 97.0%; p<0.001). However, there were no **significant changes in provision of clean bathroom and toilet (83.6 vs 80.4%; p=0.282).**	**Each health facility should assess the availability and functioning of toilets and bathrooms. Where available, they should be functional and kept clean. If lacking, they should be requested. One health centre requested and had a new toilet but no report on the impact of this improvement was presented.**
MacKeith *et al*^39^	Zambian women’s experiences of urban maternity care: results from a community survey in Lusaka	Zambia	Cross-sectional/community survey questionnaires	Examine access, coverage and quality of care in midwives run maternity service		845 were interviewed. 74% would like to see improvements overall and **18.23% would like to see better hygiene in toilets and bathrooms at health facilities.**	
Griffiths and Stephenson^41^	Understanding users’ perspectives of barriers to maternal health care use in Maharashtra, India	India	Cross-sectional/interviews	Identification of key social, economic and cultural factors influencing women’s decisions to use maternal healthcare		45 women were interviewed. Respondents identified poor-quality of services offered at government institutions to be a motivating factor for delivering at home: ‘It was safe in the house and the nurse was present to do the delivery. In government hospital, delivery room is not there. **Toilet and water facilities are not there. So I felt safe to give birth in the house’.**	Socioeconomic status was not found to be a barrier to service use when women perceived the benefits of the service to outweigh the cost, and when the service was within reasonable distance.

The table summarises the characteristics of included studies and their main findings.

WASH components are presented in bold.

WASH, water, sanitation and hygiene.

The level of satisfaction with WASH provision was reported in most studies. However, some studies reported on composite indicators of patient satisfaction and these were also noted. The papers were categorised according to the type of healthcare system, in particular, findings for maternity services were presented separately. Additionally, three papers investigated improvement interventions.

### WASH in HCF other than maternity services

Several papers reported patient dissatisfaction rates with WASH in non-maternal health service. Woldeyohanes and colleagues assessed patient satisfaction with in-patient services in Ethiopia and reported 81.5% were dissatisfied with toilet cleanliness (table 2).^25^ A study in antiretroviral treatment clinic in Ethiopia showed a lower, but significant, dissatisfaction with toilet cleanliness (35.3%).^26^ The authors highlighted the importance of maintaining good hygiene levels, especially for patients with HIV/AIDS. Ezegwui and colleagues investigated patients’ satisfaction with eye care hospital in Nigeria and found that 71.7% of patients were dissatisfied with toilet facilities (only one toilet for patients and no running tap water).^27^ A study of rural healthcare system in India highlighted the link between poor WASH provision and patient dissatisfaction, with 50% respondents reporting that in surveyed government hospitals toilets are either ‘not at all usable’ or ‘dirty needed cleaning’.^28^ In addition, 3% of health facilities did not have toilets and drinking water was available in only 55% of hospitals. The authors concluded that provision of clean toilets with privacy and safe drinking water would improve client satisfaction.^28^ While all these studies reported low patient satisfaction with WASH provision, a study in an eye care hospital in India reported high patient satisfaction with toilets (83.2%), water facilities (99.4%) and cleanliness (99.4%).^29^ Indeed, no respondent judged these as poor. However, 16.9% did not answer the toilet question. It is unclear if WASH provision was adequate in the HCF investigated as the paper was not focused on WASH, thus this information was not provided.

Khamis and colleagues investigated patient satisfaction with quality of care in an outpatient department in Tanzania using perception and expectation questions and calculating mean gap score between the two components.^30^ The study reported high overall dissatisfaction with quality of care, with a mean gap score of −2.88.^30^ The mean gap score was −0.5 and −0.67 for general cleanliness and sufficient chairs and toilets, respectively (table 2), showing a moderate level of dissatisfaction.^30^

Mohammed and colleagues assessed the responsiveness of healthcare services for insured patients in Nigeria.^31^ One of the responsiveness domains was quality of facilities, which included provision of clean toilets in the hospital. Only 42.8% of users were satisfied with the quality of facilities and low-income users reported better quality of services than high-income users.^31^ Westaway and colleagues investigated interpersonal and organisational dimensions of patient satisfaction in a diabetic clinic for black patients in South Africa and found that the most important items for satisfaction were availability of a seat and a toilet in the waiting area and cleanliness.^32^

In a study investigating quality of care and contraceptive use in Kenya, 78.5% of facilities had running water; however, facility infrastructure and patient satisfaction indicators were not associated with contraceptive use.^33^ The cost of service and toilet facilities were the main areas of dissatisfaction.

Glick investigated the reliability of exit surveys frequently used to assess patient satisfaction.^34^ The respondents’ opinions were collected and answers were compared between exit and household surveys. Courtesy bias was found to influence respondents’ answers resulting in overestimates of patient satisfaction from exit surveys. This bias was stronger for subjective questions such as treatment by staff and consultation quality compared with objective questions such as facility conditions.^34^

### WASH in maternity services

Nine out of 21 studies focused on WASH conditions specifically around maternal health services, covering antenatal, delivery and postnatal care. Srivastava and colleagues conducted a systematic review investigating determinants of women’s satisfaction with maternal healthcare in developing countries and covered all three dimensions: structure, process and outcome.^35^ A good physical environment was found to be associated with a positive assessment of the health facility. In Bangladesh, when availability of services (a composite of waiting area and time, drinking water and clean toilet) was rated good, mothers were more satisfied with care quality.^35^ Cleanliness and maintenance of hygiene were also significant determinants of satisfaction in Bangladesh, Gambia, Thailand, India and Iran. Interpersonal behaviour, specifically provider courtesy and non-abuse, were the most widely reported determinants of women satisfaction.^35^ However, other factors influenced perceived maternal satisfaction including access, cost, socioeconomic status and reproductive history.^35^

Steinmann and colleagues assessed women’s satisfaction with latrines and hand washing stations in rural India and their impact on care seeking behaviour.^36^ They reported significant discrepancies between public and private health facilities. The average number of latrines per HCF was 2.4 (1.3 in public and 3.5 in private facilities). One healthcare centre had no latrine and dedicated latrines for woman were rarely available.^36^ The mean number of hand washing stations was 2.3 (0.8 for public and 3.7 for private facilities), with two public centres lacking any hand washing facilities. WASH provision is generally acceptable in private healthcare centres but needs improvement in government facilities.^36^ Good reputation, competent and respected doctors and ability to deal with complications were the main factors influencing the choice of HCF. For ambulatory care, including child birth, WASH provision was considered less important compared with prolonged hospitalisation settings.^36^

Mbwele and colleagues investigated the quality of neonatal healthcare in Tanzania.^37^ Two per cent of mothers commented on hygiene issues and one mother suggested that improvements in hygiene were needed. Most respondents reported that the condition of toilets was as expected, while a few found them worse than expected (table 2).^37^ The main reason for primary delay was quality of treatment followed by cost of medical care, while secondary delay was due to distance from home, transport and complaints about unfriendliness of care workers. Tetui and colleagues investigated the quality of antenatal care in Uganda and reported that 74.6% of respondents were satisfied with care quality, while 70% were satisfied with cleanliness.^38^ Although data on piped water and hand washing were collected as part of the assessment, no report on WASH and patient satisfaction was provided. Infection control was a major focus and 73.4% HCF were deemed to have good infection control measures.^38^ MacKeith and colleagues assessed women’s experience of urban maternity care in Zambia and reported that 74% would like to see general improvements; however, only 18.23% clearly expressed the need for better hygiene in toilets and bathrooms.^39^

Gabrysch and colleagues reported that women criticise dirty toilets, lack of water and aseptic practices, highlighting combined shortcomings in personal interaction, medical care and hygiene.^40^ They concluded that the perceived quality of care had a major influence on care seeking behaviour.^40^ Griffiths and colleagues investigated users’ perspectives of barriers to maternal healthcare use in India through identification of key social, economic and cultural factors influencing women’s decision to seek maternal care.^41^ Quality of care and safety issues as well as lack of WASH provision were motivating women to give birth at home. A respondent stated, ‘It was safe in the house and the nurse was present to do the delivery. In the government hospital, the delivery room is not there. Toilet and water facilities are not there. So I felt safer to give birth in the house’.^41^ Socioeconomic status was not a barrier to service use when women considered the benefit to outweigh the cost, providing it was within reasonable distance.^41^ Philibert and colleagues reported that, in Burkina Faso, socioeconomic status influences patients’ expectation and satisfaction, with the poorest women more satisfied with delivery environment than the wealthiest ones.^42^ Courtesy bias leads women to respond more positively to care quality questions, which does not reflect their true opinion.^42^ Courtesy bias was more pronounced for interpersonal relationships between patients and care providers,^42^ which is in accordance with the findings of Glick (in a non-maternity setting).^34^

### Improvement interventions and accreditation in HCF

Developing accreditation standards in Ugandan hospitals was investigated by Galukande and colleagues.^43^ Accreditation items included physical infrastructure, infection control and waste management. While the majority of hospitals reported having infection control protocol in place, only half were recording needle stick injuries and vermin control.^43^ Perhaps more surprisingly, 27.5% hospitals were not tracking infection rates even for caesarean sections. In addition, the authors reported inadequate capacity to sterilise equipment in all hospitals, which would contribute to HCAI.^43^ The study reported good provision of running water but no mention of sanitation. Okwaro and colleagues investigated community perception of healthcare improvement intervention in rural Uganda.^44^ The formative research showed that many HCF (in this case malaria treatment centres) lacked running water. Following the intervention, antimalarial drug availability has improved; however, other requirements including more health workers, provision of clean water and clean toilets have not been addressed. Therefore, this intervention was not sufficient to elicit major changes or influence patients’ decision about healthcare use.^44^ Indeed, several patients continued to seek care at inadequate heath centres. The authors reported that the main limitation of such an intervention is the focus on a particular disease and therefore failing to address multiple inadequacies observed in HCF in LMIC.

One paper investigated a criteria-based audit to improve a maternity unit in Malawi, where an initial audit resulted in the formulation of recommendations and a second audit 3 months later would report on any observed improvements.^45^ Significant improvements in cleanliness were achieved post audit; however, no significant changes in provision of clean toilets and bathrooms were noted.^45^ The authors reported that one health facility requested and obtained a new toilet, which should contribute to address the issue of inadequate WASH provision in healthcare setting.^45^

## Discussion

**Figure 2:**
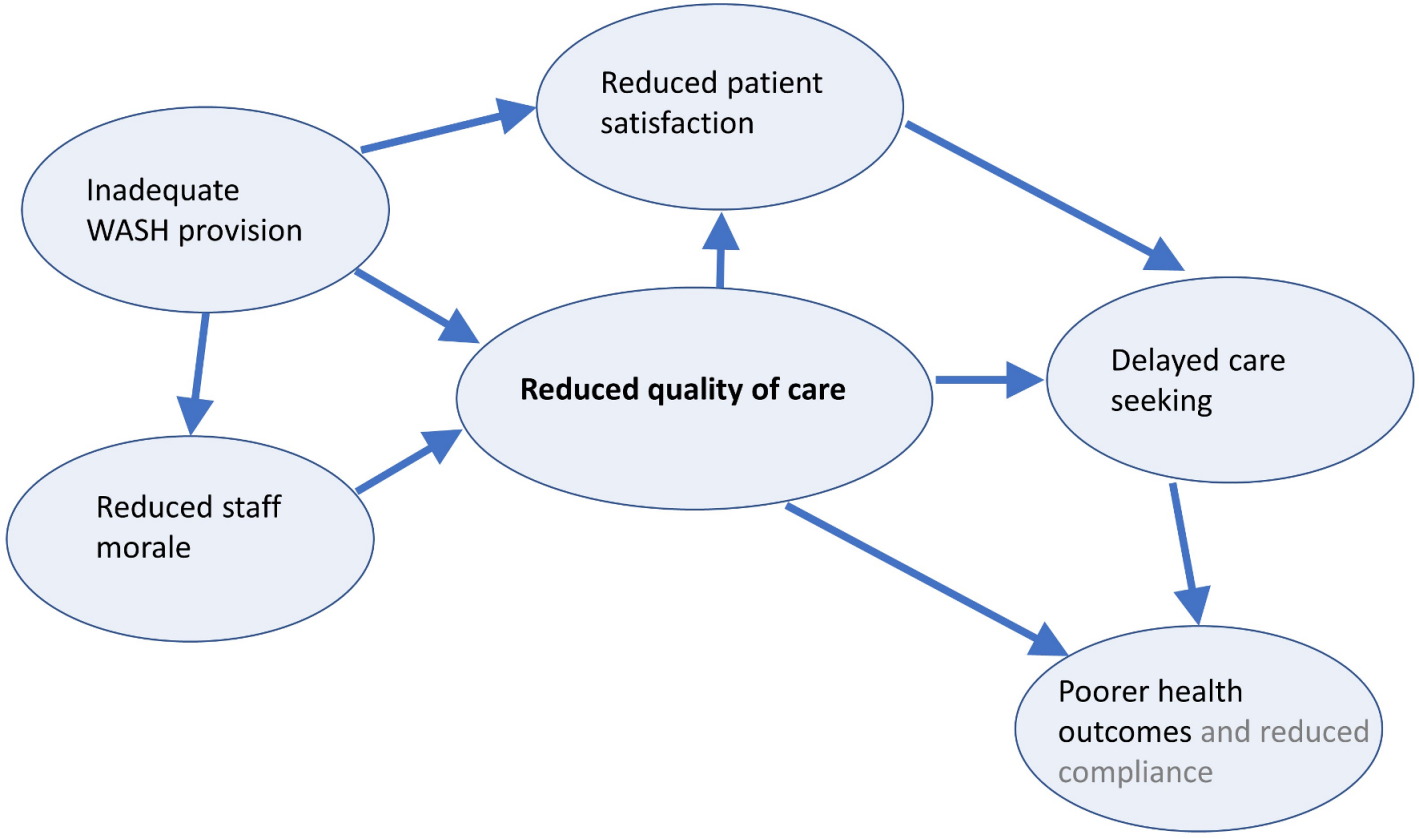
Conceptual model of implications of patient dissatisfaction with care quality. The model details the interactions between patient dissatisfaction, inadequate WASH provision, care seeking behaviour and health outcomes. WASH, water, sanitation and hygiene.

Patient satisfaction is a good indicator of quality of care provided and impacts on care seeking behaviour. In the reviewed studies, inadequate WASH in HCF was associated with increased patient dissatisfaction and was even a barrier to service use in some settings (most notably maternity services). This systematic review of current evidence has informed a conceptual model of patient dissatisfaction, detailing relevant factors and repercussions of low patient satisfaction (figure 2). In this model, patient dissatisfaction results in delayed care seeking, poor health outcomes and reduced staff morale. Good infrastructure including adequate WASH provision is an integral part to high quality of healthcare. Inadequate WASH provision is one of the elements influencing patient dissatisfaction, though it was not found to be a major driver. Other factors relevant in resource-poor settings were significantly influencing patient satisfaction and care seeking behaviours in LMIC. The relative importance of WASH on patient satisfaction is context-specific and depends on the type of healthcare service and the length of stay. Indeed, the lack of safe WASH facilities in delivery rooms was frequently cited as the reason for women to prefer home delivery. Women expect HCF to have adequate WASH, and rightly so, as this is pivotal for their human right, dignity and infection prevention. Achieving this, however, remains a distant prospect in many healthcare settings in LMIC.

The limitations of this study include a relatively small publication window (2000–2016), which was chosen to exclude historic (or outdated) WASH provision and a search strategy that could have been further optimised to retrieve all relevant papers. Potential further limitations are the difficulty of retrieving eligible LMIC research, likely to be published in national journals not indexed in the databases searched and studies are not necessarily indexed properly (particularly regarding LMIC status/country affiliation). Finally, the studies included were mostly cross-sectional with potentially biased outcome measures and perhaps more importantly no study designed to specifically assess the causal effect of WASH provision on patient satisfaction and/or care seeking behaviour was found. The limitations of some of the included studies are related to study design, such as small sample size, lack of randomisation and patient recruitment procedures, as well as outcome measures such as heterogeneous indicators of patient satisfaction and potentially biased findings.

This review focused on WASH and patient satisfaction/care seeking because of the large disease burden associated with delayed care seeking. The link between perceived quality of care and attendance at HCF (patients who received quality care tend to return and recommend the facility to relatives) was supported by several studies and the WHO recommends the evaluation of patients’ satisfaction for the improvement of HCF.^46^ However, perceived quality of care is highly subjective. It includes satisfaction with the outcome, the interventions and the service received (staff friendliness, availability of supplies and waiting times) as well as objective measures of care quality such as facility infrastructure, equipment and staffing.^40^ However, even these measures are subjective because they depend on the discrepancy between expectation and reality, strongly influenced by socioeconomic traits and subpopulation groups. Indeed, it was reported that wealthier women and patients with higher education were consistently less satisfied with delivery environment and quality of care, respectively.^31 42^ It was noted, however, that factors other than WASH actually drive the selection and use of health facility.^36^ Therefore, it is perhaps not surprising that patients continue to use HCF with inadequate WASH provision (table 2).^44^

The evaluation of patient satisfaction is usually performed using patient questionnaires, administered at either the HCF or households. It has been shown that exit questionnaires tend to overestimate patient satisfaction level due to courtesy bias (although this was mainly for treatment by staff and consultation quality and not facility condition).^34^ Intimidation bias was also reported when female interviewees felt intimidated by a male interviewer.^42^ Therefore, household surveys may provide more reliable estimates of patient satisfaction.^34^ However, household surveys could also yield biased results as they are associated with substantial under-reporting of healthcare use, especially when the recall period was over 1 month.^47^

The availability of skilled birth attendants is crucial to provide emergency obstetric care and reduce maternal and newborn mortality.^48^ This is part of the official guidance and improving WASH provision should increase use of maternal health services in LMICs. Concernedly, a study reported higher mortality rates after obstetric care.^49^ The reasons were: seeking help very late and in critical condition and lack of timely and adequate care once at the health facility. Birth attendants may not provide socioculturally appropriate and respectful care leading to poor uptake.^48^ Previous delivery by a male provider made women choose home delivery during the subsequent pregnancy (OR 3.90; 95% CI 2.30 to 6.65).^46^ It was stated that ‘efforts aimed at improving maternal and child health in developing countries should take cognisance of the sociodemographic and cultural underpinnings of maternal health seeking behaviour’.^50^ Complaints of abuse, neglect and poor treatment are common in maternity services.^51^ Therefore, in addition to improving facilities’ infrastructure, care quality and cost-effectiveness, improvements in maternity services should also address providers’ attitudes and interpersonal behaviours.^48^ This highlights the scale and complexity of the issues investigated and the high number of shortcomings that need to be addressed.

The importance of WASH in HCF extends beyond patient satisfaction and care seeking behaviour because inadequate WASH may also be associated with a significant burden of HCAI. Poor sanitary conditions and hand hygiene in hospital settings would result in several gastrointestinal and opportunistic infections. Unfortunately, poor hand washing practices around birth are still prevalent and continue to pose risks to mother and baby. In an observational study, the proportion of birth attendants who washed their hands prior to assisting with delivery was 24% in India, 69% in Bangladesh and 32% in Nepal.^52^ Hand washing of birth attendants was associated with 49% reduction in maternal mortality (OR 0.51, 95% CI 0.28 to 0.93)^52^ and 19% (range 1%–34%) reduction in all cause neonatal mortality.^53^ Effective hand washing in HCF has benefits for a wide range of other HCAI,^54^ although adherence to good hand hygiene practices is a persistent challenge. Addressing this issue requires changes in both behaviour and infrastructure; hand hygiene practices will only improve if healthcare workers are motivated to change their behaviour and when adequate facilities (taps with running water and soap) are available.

## Conclusion

The provision of adequate WASH in HCF is important to protect vulnerable populations and reduce HCAI. However, WASH provision is still inadequate in many HCF in LMIC. This systematic review assessed the impact of WASH provision on care seeking behaviour and patient satisfaction. Our review suggests that improving WASH conditions will decrease patience dissatisfaction, which may increase care seeking behaviour and improve health outcomes but that more rigorous research is needed.
